# The Frequency of Cutaneous Manifestations in Hepatitis C: A Cross-sectional Study in a Tertiary Care Hospital in Pakistan

**DOI:** 10.7759/cureus.6109

**Published:** 2019-11-09

**Authors:** Saleh Mohammad, Bashir Chandio, Aftab A Soomro, Salma Lakho, Zamanat Ali, Faizan Shaukat

**Affiliations:** 1 Gastroenterology, Ghulam Muhammad Mahar Medical College and Hospital, Sukkur, PAK; 2 Internal Medicine, Ghulam Muhammad Mahar Medical Hospital, Sukkur, PAK; 3 Pathology, Ghulam Muhammad Mahar Medical Hospital, Sukkur, PAK; 4 Internal Medicine, Ghulam Muhammad Mahar Medical College and Hospital, Sukkur, PAK; 5 Internal Medicine, Jinnah Postgraduate Medical Centre, Karachi, PAK

**Keywords:** hepatitis c, dermatology, skin manifestation

## Abstract

Introduction

Even though the liver is the main targeted organ in hepatitis C, the manifestations of the disease are not limited to hepatic involvement. Other tissue types are often involved as well. Hepatitis C has dermatological and mucocutaneous manifestations.

Methods

This study was conducted from May 2016 to April 2017 in the gastroenterology unit of Ghulam Muhammad Mahar Medical College, Sukkur, Sindh, Pakistan. All participants with a clinical diagnosis of hepatitis C with cutaneous manifestations were included in the study after securing informed consent. Demographic data and detailed cutaneous examination results with full morphological descriptions were recorded in patient-completed questionnaires.

Results

Of the 212 participants, 141 (66.6%) were males and 71 (33.4%) were females. The mean age was 32 (±9) years (range: 17-58 years). The most common cutaneous manifestation was pruritus (33.96%), followed by lichen planus (LP) (23.5%).

Conclusion

Physicians should try to recognize extrahepatic manifestations of a hepatitis C infection as it may help in early diagnosis efforts. While managing hepatitis C, we recommend a multidisciplinary approach to tackle cutaneous and other extrahepatic manifestations.

## Introduction

Approximately 130-170 million people are infected by the hepatitis C virus (HCV) around the world, and it is responsible for approximately 350,000 deaths every year [1-3]. HCV infection is a major healthcare problem in Pakistan, with acute and chronic infections responsible for liver damage, cirrhosis, and hepatocellular carcinoma. More than 11% of the population is infected with HCV in Pakistan. The most common genotype in Pakistan is HCV genotype 3a [4]. While the liver might be the main target in hepatitis C, the manifestations of the disease are not limited to hepatic involvement, and other tissues are involved as well, particularly dermatological and mucocutaneous tissues. [5] Recognizing extrahepatic manifestations of HCV may help in early diagnosis efforts [5].

Various data are available in the literature regarding the association of chronic HCV infection with a wide spectrum of cutaneous manifestations. Common skin diseases with strong epidemiological and pathogenetic associations with chronic HCV infection are mixed cryoglobulinemia, lichen planus (LP), and porphyria cutanea tarda (PCT) [5]. Other manifestations may include pruritus, psoriasis, vitiligo, and urticaria [5].

Unfortunately, there are very limited data available on cutaneous manifestations in HCV, particularly from rural areas. In this study, we aim to determine the frequency of cutaneous manifestations of HCV infection.

## Materials and methods

This cross-sectional descriptive study was conducted from May 2016 to April 2017 in the gastroenterology unit of Ghulam Muhammad Mahar Medical College, Sukkur, Sindh, Pakistan. All participants with a clinical diagnosis of HCV with cutaneous manifestation were included in the study after securing written informed consent. Patients with other chronic diseases such as diabetes, liver disease due to reasons other than HCV, and chronic renal disease were excluded. Participants who had skin disease prior to the diagnosis of hepatitis C and pregnant women were also excluded.

Demographic data and a detailed cutaneous examination that included full morphological descriptions were recorded in patient-completed questionnaires. Diagnoses were made with the help of a dermatologist. Necessary investigations such as skin biopsy, serum cryoglobulins, ultrasound, albumins, prothrombin time, and complete blood count were conducted when needed. Palmar erythema, jaundice, spider nevi, and telangiectasia were considered as cutaneous manifestations of chronic liver disease for the purpose of our study.

Data were analyzed using IBM SPSS Statistics for Windows, Version 21.0 (IBM, Armonk, NY). Mean and standard deviation (SD) were calculated for continuous variables such as age. Frequencies and percentages were calculated for categorical variables including gender and cutaneous manifestation.

## Results

A total of 212 patients [141 males (66.6%), 71 females (33.4%)] met the inclusion criteria and were diagnosed with cutaneous manifestations of HCV. The mean age was 32 (±9) years (range: 17-58 years). The most common cutaneous manifestation was pruritus (33.96%), followed by LP (23.5%). Some participants had more than one cutaneous manifestation (Table 1).

**Table 1:** Cutaneous manifestations by type

Cutaneous manifestation	Number of patients
Pruritus	72 (33.96%)
Lichen planus	50 (23.5%)
Cryoglobulinemia	18 (8.49%)
Urticaria	14 (6.6%)
Vitiligo	8 (3.77%)
Erythema nodosum	4 (1.88%)
Necrolytic acral erythema	2 (0.94%)
Porphyria cutaneous tarda	2 (0.94%)

There were 27 (12.7%) patients with a cutaneous manifestation of chronic liver disease. The most common cutaneous sign in patients with chronic liver disease was palmar erythema (4.7%; Table 2).

**Table 2:** Cutaneous manifestations in patients with chronic liver disease

Cutaneous manifestation in chronic liver disease	Number of patients (%)
Palmar erythema	10 (4.7%)
Jaundice	8 (3.77%)
Spider nevi	2 (0.94%)
Telangiectasia	2 (0.94%)

## Discussion

The mechanism behind cutaneous manifestations in patients with hepatitis C is not well understood. However, there are several hypotheses to explain this phenomenon [6]. One of these hypotheses states that chronic HCV infection results in hyperstimulation of the immune system, involving both the humoral and cellular immunity. This initiates a broad-based immunological response causing monoclonal and polyclonal expansion of B cells and T cells [7]. These massive immune responses against self-antigens are a major cause of extrahepatic manifestations including cutaneous infections. Another possible explanation for cutaneous manifestations of HCV infection is the pathogenic mimicry of HCV particles found in many damaged tissues, including oral LP lesions. Given their structural resemblance to the normal tissue antigens, antibodies against HCV antigens cross-react with normal tissue antigens and initiate the disease process [5,8].

We found that 87.3% of patients with HCV infection had cutaneous manifestation, which is a much higher prevalence than noted in other studies. This could be explained by the more persistent genotype in our population [4]. A local study conducted in 2016 reported skin manifestations in 54% of their patients [9]. A study conducted in Egypt reported that 45.8% of HCV-infected patients presented with various cutaneous infections [10].

In our study, pruritus was the most frequent skin disorder, seen with a frequency of 33.6%, which is higher than other reports in the literature. However, pruritus was the most common cutaneous manifestation, with a frequency of 15.9%, in one of the local studies. A study conducted in Egypt has reported a frequency of 21.3%. Also, Dervis and Serez reported a frequency of 18.57% in a study conducted in Turkey [9-11].

In the current study, LP occurred with a frequency of 23.5%. Shahzadi et al. reported that one-third of patients presenting with LP were positive for HCV infection [12]. According to a meta-analysis in which 19 studies were analyzed to determine a correlation between HCV and LP, patients with LP should be regularly screened for HCV seropositivity [13].

Cryoglobulinemia is a cutaneous manifestation of cold-sensitive antibodies called cryoglobulins, and it was present in 8.49% of our study population. According to the literature from studies in developed countries such as Australia and the US, cryoglobulinemia is observed in nearly half of chronic hepatitis C patients [14,15]. Other skin manifestations observed in patients with HCV infection included urticaria, vitiligo, erythema nodosum, necrolytic acral erythema, and porphyria cutaneous tarda.

In our study, 12.7% of patients with HCV infection developed chronic liver disease. The most common cutaneous manifestation these patients developed was palmar erythema (4.7%). This relationship between chronic liver disease and skin manifestation is well-established [16,17].

The study has some limitations. Firstly, it was a single-center study and the result cannot be generalized to the entire population. Secondly, since it was a cross-sectional study, a strong association could not be established between skin manifestation and Hepatitis C. Further large- scale, multi-center prospective studies are required to better understand the prevalence and implications of cutaneous manifestations in Hepatitis C.

## Conclusions

Pruritus, LP, and cryoglobulinemia were the most common skin manifestations in patients with hepatitis C. Cutaneous manifestations accompanying HCV infection place a substantial burden on public healthcare and have detrimental effects on patients. This study highlights the need for HCV screening of patients presenting with various cutaneous disorders to provide early diagnosis and treatment for HCV infection and prevent further transmission of the disease. It is important to understand dermatological conditions associated with hepatitis C as it may help in early diagnosis and management of hepatitis C.

## References

[1] Saadoun D, Asselah T, Resche-Rigon M (2006). Cryoglobulinemia is associated with steatosis and fibrosis in chronic hepatitis C. Hepatology.

[2] Cacoub P, Gragnani L, Comarmond C, Zignego AL (2014). Extrahepatic manifestations of chronic hepatitis C virus infection. Dig Liver Dis.

[3] Wasley A, Alter MJ (2000). Epidemiology of hepatitis C: geographic differences and temporal trends. Semin Liver Dis.

[4] Arshad A, Ashfaq UA (2017). Epidemiology of hepatitis C infection in Pakistan: current estimate and major risk factors. Crit Rev Eukaryot Gene Expr.

[5] Garcovich S, Garcovich M, Capizzi R, Gasbarrini A, Zocco MA (2015). Cutaneous manifestations of hepatitis C in the era of new antiviral agents. World J Hepatol.

[6] Schwartz RA, Birnkrant AP (2019). Cutaneous manifestations of hepatitis C. Medscape.

[7] Muzaffar F, Hussain I, Haroon TS (2016). Hepatitis C: the dermatologic profile. J Pakistan Assoc Dermatol.

[8] Dedania B, Wu GY (2015). Dermatologic extrahepatic manifestations of hepatitis C. J Clin Transl Hepatol.

[9] Azfar NA, Zaman T, Rashid T, Jahangir M (2016). Cutaneous manifestations in patients of hepatitis C. J Pakistan Assoc Dermatol.

[10] Raslan HM, Ezzat WM, Abd El Hamid MF, Emam H, Amre KS (2009). Skin manifestations of chronic hepatitis C virus infection in Cairo, Egypt. East Mediterr Health J.

[11] Dervis E, Serez K (2005). The prevalence of dermatologic manifestations related to chronic hepatitis C virus infection in a study from a single center in Turkey. Acta Dermatovenerol Alp Pannonica Adriat.

[12] Shahzadi N, Altaf F, Raffad Raffad, Anjum R, Saeed W, Butt G (2019). Association of hepatitis C virus with various forms of lichen planus. J Pakistan Assoc Dermatol.

[13] Alaizari NA, Al‐Maweri SA, Al‐Shamiri HM, Tarakji B, Shugaa‐Addin B (2016). Hepatitis C virus infections in oral lichen planus: a systematic review and meta‐analysis. Aust Dent J.

[14] Lunel F, Musset L, Cacoub P (1994). Cryoglobulinemia in chronic liver diseases: role of hepatitis C virus and liver damage. Gastroenterology.

[15] Cicardi M, Cesana B, Del Ninno E, Pappalardo E, Silini E, Agostoni A, Colombo M (2000). Prevalence and risk factors for the presence of serum cryoglobulins in patients with chronic hepatitis C. J Viral Hepat.

[16] Satapathy SK, Bernstein D (2011). Dermatologic disorders and the liver. Clin Liver Dis.

[17] Berk DR, Mallory SB, Keeffe EB, Ahmed A (2007). Dermatologic disorders associated with chronic hepatitis C: effect of interferon therapy. Clin Gastroenterol Hepatol.

